# Glucocorticoids Shape Macrophage Phenotype for Tissue Repair

**DOI:** 10.3389/fimmu.2019.01591

**Published:** 2019-07-09

**Authors:** Thibaut Desgeorges, Giorgio Caratti, Rémi Mounier, Jan Tuckermann, Bénédicte Chazaud

**Affiliations:** ^1^Institut NeuroMyoGène, Université Claude Bernard Lyon 1, Univ Lyon, CNRS UMR 5310, INSERM U1217, Lyon, France; ^2^Institute of Comparative Molecular Endocrinology, University of Ulm, Ulm, Germany

**Keywords:** glucocorticoids, macrophages, inflammation, tissue repair, phagocytosis glucocorticoid receptor

## Abstract

Inflammation is a complex process which is highly conserved among species. Inflammation occurs in response to injury, infection, and cancer, as an allostatic mechanism to return the tissue and to return the organism back to health and homeostasis. Excessive, or chronic inflammation is associated with numerous diseases, and thus strategies to combat run-away inflammation is required. Anti-inflammatory drugs were therefore developed to switch inflammation off. However, the inflammatory response may be beneficial for the organism, in particular in the case of sterile tissue injury. The inflammatory response can be divided into several parts. The first step is the mounting of the inflammatory reaction itself, characterized by the presence of pro-inflammatory cytokines, and the infiltration of immune cells into the injured area. The second step is the resolution phase, where immune cells move toward an anti-inflammatory phenotype and decrease the secretion of pro-inflammatory cytokines. The last stage of inflammation is the regeneration process, where the tissue is rebuilt. Innate immune cells are major actors in the inflammatory response, of which, macrophages play an important role. Macrophages are highly sensitive to a large number of environmental stimuli, and can adapt their phenotype and function on demand. This change in phenotype in response to the environment allow macrophages to be involved in all steps of inflammation, from the first mounting of the pro-inflammatory response to the post-damage tissue repair.

Macrophages therefore, appear to be an ideal target of anti-inflammatory drugs due to their central role in inflammation. Glucocorticoids (GCs) are highly potent anti-inflammatory drugs, commonly used around the world. GCs have been used for decades to treat a variety of inflammatory diseases such as rheumatoid arthritis, contact allergy, or pulmonary diseases. Since the first GC therapies during the 1950s, various synthetic GCs have been developed to optimize their action, and new molecules are still under development to modulate therapeutic effects vs. the adverse effects of these drugs. Surprisingly, given the importance of macrophages in the inflammatory response, the direct effects of GCs on macrophages are less well-documented. The present review aims at summarizing the knowledge on macrophage functions during the post-injury inflammatory response, with a focus on sterile inflammation and tissue repair, discussing how GC signaling pathways operate in macrophages, and finally on the specific action of GCs on macrophages.

## Macrophages and Tissue Repair—Example of Skeletal Muscle Regeneration

### Similar Macrophage Subtypes Are Found in Various Tissues During Repair

Macrophages belong to the innate immune system, however their role is far more than protecting against pathogens. In the late nineteenth century, Metchnikoff originally described and named these cells as “macro” (big) “phage” (eaters) due to their phagocytotic activity. In the following 100 years, scientists discovered that macrophages are not only phagocytic cells. Different macrophage subtypes were described, first in *in vitro* experiments, based on the main cytokinic activation of lymphocytes, allowing macrophages to be divided into different categories. “Classically activated” macrophages are induced by stimulation with the Th1 cytokine IFNγ and “alternatively activated” macrophages, involved in anti-inflammatory processes were observed when using the Th2 cytokine IL-4 (1). These two activation states were also called M1 (or pro-inflammatory macrophages) and M2 (or anti-inflammatory macrophages), respectively. However, this simplistic view of two potential statuses was quickly expanded on. Macrophages can adopt a very large panel of phenotypes depending on the inflammatory cues they encounter, even *in vitro* (2–4). *In vivo*, the situation is more complex. The terms M1 and M2, although widely used, are not appropriate to describe specific and dynamic inflammatory status that occurs in the inflammatory milieu of a living organism (5, 6). The Ly6C (Lymphocyte antigen 6 complex, a membrane protein expressed by monocytes, and macrophages) and CX3CR1 (chemokine (C-X3-C motif) receptor 1, another transmembrane protein involved in the adhesion and migration of leukocytes) antigens have been widely used to classify pro-inflammatory and anti-inflammatory macrophages in the context of post-injury inflammatory response (7). During sterile inflammation, pro-inflammatory Ly6C^pos^CX3CR1^neg^(CCR2^pos^F4/80^low^) cells infiltrate the injured tissue. After a rather undefined set of signaling events, a phenotypic switch occurs whereby macrophages lose Ly6C and CCR2 and gain CX3CR1 and F4/80 (forming Ly6C^neg^CX3CR1^pos^CCR2^low/neg^F4/80^high^ cells) corresponding to their anti-inflammatory status (8). This sequence of events from the infiltration of pro-inflammatory macrophages to the phenotypic switch toward anti-inflammatory activity appears to be universal. These events have been described after injury in heart (9), central nervous system (10, 11), liver (12), kidney (13, 14), and skeletal muscle (15–18).

### Skeletal Muscle Regeneration

**Figure 1:**
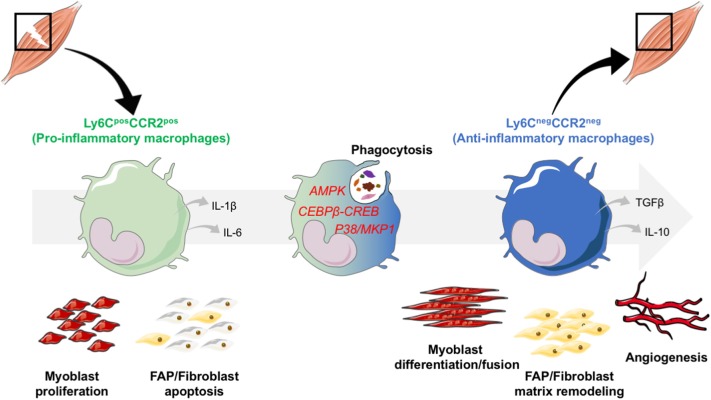
Phenotype switch of macrophages regulates skeletal muscle regeneration. After an injury, monocytes are recruited from the bloodstream, and infiltrate the damaged area. In the tissue, monocytes acquire a damaged associated pro-inflammatory phenotype. They secrete inflammatory cytokines such as IL-1β and IL-6 and exert specific functions: they stimulate the proliferation of the myogenic precursors (myoblasts) and trigger fibroblast apoptosis to avoid excessive matrix deposition. Upon phagocytosis of cell debris that triggers the activation of AMPK, CEBPβ-CREB axis and P38/MKP1 pathways, pro-inflammatory macrophages switch their phenotype toward an anti-inflammatory restorative phenotype. Through the secretion of a variety of factors, among which anti-inflammatory cytokines IL-10 and TGFβ, anti-inflammatory macrophages are involved in tissue repair and regeneration through the stimulation of myoblast differentiation and fusion, of FAP/fibroblasts for matrix remodeling and of angiogenesis.

The core cell type within skeletal muscle is the myofiber—a multinucleated cell formed by fusion of precursor cells (19). Skeletal muscle has a high regenerative capacity, after injury, muscle regenerates *ad integrum*, where the old damaged cells are replaced by proliferation and differentiation of satellite cells, which are the muscle resident stem cells (MuSCs). Skeletal muscle regeneration, therefore, is an ideal paradigm to study the biological events involved in tissue repair/regeneration, helped by highly reproducible experimental models in mouse (20). Satellite cells are localized under the basal lamina surrounding each myofiber, in a quiescent state. After an injury, damaged myofibers undergo necrosis which triggers alteration of the satellite cell niche, in turn leading to their activation (19). Activated MuSCs proliferate, in order to produce a critical pool of cells necessary to repair muscle, after which MuSCs differentiate into myocytes, that eventually fuse to form new myofibers. While myogenesis takes place, multiple other biological processes occur simultaneously during muscle regeneration. Angiogenesis is required for efficient muscle regeneration. Endothelial cells and MuSCs communicate through secreted factors to mutually promote myogenesis and angiogenesis (21). Fibro-Adipogenic Precursors (FAPs) control the extracellular matrix remodeling during muscle regeneration, depending on the number and differentiation status of the FAPs (22). Thus, muscle regeneration is a complex process where multiple cell types interact and coordinate to reconstruct the tissue (Figure 1).

Each step of muscle regeneration is linked to the inflammatory response, which is mainly mediated by macrophages. Macrophages modulate myogenesis through MuSCs (17), as well as angiogenesis (21), and matrix remodeling (22) that occur concomitantly. Macrophages represent more than 75% of the leukocytes present in a regenerating muscle; however other immune cells are present in lower numbers (16) and are more prominent during the early steps of muscle regeneration. Neutrophils are transiently present during the very first days after injury, but their contribution to muscle regeneration has not been deciphered yet and may depend on the extent of the injury (23). Eosinophils participate in muscle regeneration through the secretion of IL-4 that activates FAP proliferation (24). Tregs secrete the growth factor amphiregulin that stimulates MuSC expansion and differentiation (25). Therefore, macrophages are major actors in the regulation of skeletal muscle regeneration through the establishment of various interactions with several cell types. While the above-mentioned studies clearly show how macrophagic populations impact on other cell types, the effect of those cells on macrophage phenotype and function has not been evidenced yet.

### The Inflammatory Phase During Muscle Regeneration

Tissue injury triggers the release of chemoattractants into the bloodstream that recruit circulating leukocytes. Monocyte entry into the injured muscle is regulated through the CCL2 (MCP1)/CCR2 axis. In mouse models of CCR2 or CCL2 depletion, muscle regeneration is severely hindered (26, 27). Indeed, only circulating Ly6C^pos^CCR2^pos^ monocytes are recruited into the injured muscle (6, 15, 18). In the *nur77*KO mouse model where CCR2^neg^Ly6C^neg^ monocytes are absent from the circulation, muscle regeneration occurs normally, indicating that circulating CCR2^neg^Ly6C^neg^ monocytes are not recruited into the injured muscle (15, 18). Once in the tissue, macrophages clear debris from apoptotic and necrotic cells through efferocytosis. They also potentiate the survival and growth of MuSCs by establishing direct cell-cell contacts (28, 29). Moreover, pro-inflammatory macrophages secrete factors such as IL-6, IL-1β, or VEGF that stimulate MuSC proliferation (15, 17). Finally, pro-inflammatory macrophages control FAP apoptosis, preventing excess matrix deposition by fibroblastic cells (22, 30).

### Macrophage Phagocytosis and the Resolution of Inflammation

At the time of resolution of inflammation, pro-inflammatory macrophages shift toward an anti-inflammatory phenotype (Figure 1). Signaling pathways involved in this switch are beginning to be documented in the literature. Currently, 3 main intracellular pathways have been described: AMPK, p38/MKP1, CREB-C/EBPβ (see below section “Time and space orchestration of the inflammatory response”). While the activation of these pathways is required, the activating upstream cues are still unknown. However, one likely candidate is the phagocytotic pathway that has been shown to be essential for the acquisition of an anti-inflammatory phenotype. Efferocytosis, that is the ingestion of apoptotic cells by macrophages, results in a reduction of pro-inflammatory markers, and an increase in the expression of anti-inflammatory markers, suggesting that the death signals of apoptotic cells may contribute to the generation of an anti-inflammatory phenotype (31–33). Anti-inflammatory macrophages act on several cell types in regenerating skeletal muscle, inducing both differentiation, and fusion of MuSCs as well as growth of the newly regenerated myofibers (15–17). Anti-inflammatory macrophages promote extracellular matrix remodeling by inducing fibroblast survival and collagen production through the secretion of TGF-β (30). *In vitro* experimentation has shown that anti-inflammatory macrophages stimulate endothelial cell sprouting and differentiation, inducing vessel formation concomitantly to myogenesis, through the secretion of specific effectors, such as the cytokine Oncostatin M (21). Accordingly, CCR2 KO mice exhibit defect of vascularization in the regenerating muscle, as macrophages are not efficiently recruited to the site of injury (34). Thus, anti-inflammatory macrophages are a key component of the regeneration phase. They act on multiple cell types within the muscle, promoting growth of newly formed muscle cells, remodeling of extracellular matrix and revascularization all simultaneously, allowing the full, and importantly functional, recovery of the muscle tissue.

### Time and Space Orchestration of the Inflammatory Response

The inflammatory response needs to be tightly orchestrated to be efficient, and the regulation of macrophage activity is no exception. Resolution of inflammation is a key step in skeletal muscle regeneration, that must occur timely. Indeed, when the pro-inflammatory phase is blunted by the inhibition of the expression of the pro-inflammatory cytokine IFNγ (35) or reduced by the early administration of anti-inflammatory cytokine IL-10 (36), muscle regeneration is impaired, resulting in the formation of smaller myofibers. Similarly, blunting the inflammatory phase by administrating anti-inflammatory drugs or icing the early injured muscle to prevent the entry of monocytes is detrimental for muscle regeneration [reviewed in (37)].

AMP-activated protein kinase (AMPK), a key metabolic regulator is also important for the generation of anti-inflammatory macrophages (16). Similarly, the p38/MKP1 pathway (MAP kinase pathway) modulates the phenotype of macrophages. Inhibition of the phosphatase MKP1 allows for an early activation of AKT, leading to a too early acquisition of the anti-inflammatory status in macrophages, resulting in to an impairment of muscle regeneration (36). Finally, blocking the CREB-C/EBPβ cascade prevents the acquisition of the anti-inflammatory phenotype of macrophages, that also impairs muscle regeneration (38). Given the importance of the process of the resolution of inflammation for tissue homeostasis, it is likely that other pathways are also involved in the switch of the inflammatory status of macrophages.

## Glucocorticoids: a General Overview

### Origins of GCs

The hypothalamic-pituitary-adrenal axis is critical for the regulation of a variety of biological processes: stress, feeding, circadian rhythm, growth, and reproduction. GC production is regulated, via multiple hormonal inputs at all levels of the axis [reviewed in (39, 40)]. The hypothalamus secretes corticotropin releasing hormone (CRH), the first step in the regulation of GC secretion. CRH is controlled through input of the nervous system, such as exposure to stress, circulating hormones like progesterone and adrenaline, but also by GCs. CRH acts on the pituitary gland to induce the secretion of the Adreno Cortico Tropic Hormone (ACTH) into the bloodstream. ACTH binds to its receptor on cells of the adrenal cortex to regulate the secretion of a variety of hormones, especially the GC cortisol (in humans), and corticosterone (in mouse). The HPA axis, and therefore GC production is also under control of the inflammatory response. Using computational modeling and comparison to clinical data, it was demonstrated that after an inflammatory trigger, ACTH and cortisol rise within minutes to hours, slightly after cytokine release. However, this is not maintained for long, and returns to baseline after 10 h (41). The homeostatic release of GCs after an inflammatory challenge plays an important protective role, which without (e.g., through a disrupted HPA axis) results in relatively mild inflammation becoming deadly [reviewed in (42)]. Investigation into the potential medical use of GCs started in the 1930s, where Philip Hench, Edward Kendall, and Tadeusz Reichstein showed the incredible therapeutic potential of these molecules as anti-inflammatory drugs, and later received the Nobel prize for their work in 1950. From that point, GC therapies spread all around the world and are still used today to counter inflammation.

### The GC Receptor

GCs act through the Glucocorticoid Receptor (GR), a member of the nuclear receptor superfamily, and first cloned in 1985 (43). The gene encoding GR is located on the locus 5q31.3 in the human genome comprised of 9 exons (43). GR expression gives rise to the expression of 2 major isoforms: GRα (777 amino acids) and GRβ (742 amino acids), along with other less well-expressed (and less well-studied) isoforms (43). GRα is the active isoform that binds GCs and that regulates target gene expression. GRβ isoform is a regulator of the α isoform, acting as a dominant negative (44, 45). A third isoform of the receptor, GRγ has also been characterized. This isoform only differs from GRα by one arginine in the DNA Binding Domain (DBD) that alters the capacity for the isoform to regulate gene expression, giving GRγ its own transcriptomic profile (46). This altered profile may play a role in GC resistant leukemia (47), however its action during inflammation has not yet been extensively studied.

The 3D structure of GR is comprised of several domains: the N-terminal domain, the DBD, the hinge region, the Ligand Binding Domain (LBD) and the C-terminal domain (48–50).

GR, like other nuclear receptors is a ligand regulated transcription factor, which regulates gene expression by binding either directly, or indirectly to the genome [review in (51)]:

- Activation: after ligand binding in the cytoplasm, GR translocates to the nucleus, and directly binds specific palindromic regions on DNA called Glucocorticoid Response Elements (GREs). GREs are present in the regulatory regions, such as the promoters, enhancers, and even within the exons or introns of target genes (such as *Gilz* and *Dusp1*) and binding of GR dimers induces the transcription of these genes (positive GRE) (51). Transactivation can also occur by a tethering mechanism, whereby GR associates with other transcription factors that positively drive gene expression. Transcription can also be induced by monomeric GR that binds DNA to a half-site motif (52).
- Repression: as with activation, nuclear GR can bind DNA and represses the transcription of genes. GR can directly act as a monomer in association with other transcription factors such as NFκB (53) or AP-1 (54) to transrepress gene expression by a tethering mechanism (51). GR monomer sequestrates transcription factors to prevent their binding to promoters and so to prevent transcription. Moreover, GR cis-repress genes by directly binding so called negative GREs or by directly binding the NFκB or AP-1 response elements (55). More mechanisms are currently emerging driven by genome wide studies that are reviewed in detail elsewhere in this Research Topic (Escoter-Torres et al., Submitted).

Thus, GR is a transcription factor that regulates gene expression through several pathways [reviewed in (45, 49, 56, 57)] and in a tissue dependent manner (58). Non-genomic effects of GCs, that is GC regulated actions that are independent from the regulation of gene expression, have been described in several tissues and that were very recently reviewed in Panettieri et al. (59).

### Adverse Effects of GCs

During the 1960s, it became clear that clinical use of GCs causes severe metabolic side effects. In 1970, David and colleagues reviewed 20 years of GC utilization (60). They discussed side effects that were observed in almost all tissues of the body. In 1970, it was already known that long exposure to GCs was responsible for several metabolic disturbances, but more recent studies have expanded on this, dramatically enhancing our knowledge about GC effects on metabolic organs. Chronic GC use results in the development of type 2 diabetes (due to increased gluconeogenesis, hepatosteatosis, decreased insulin sensitivity, and decreased glucose consumption) (61–63), skin (64, 65), and muscle atrophy (66), and bone mass reduction (both due to induction of catabolism and/or reduction of anabolism) (67). Moreover, free fatty acids are increased in the bloodstream and in clinical cases of GC excess—for example Cushing's Disease, this results in increased adipose tissue mass, but usually localized to the face and truck, resulting in a “Moon-Face” and “Buffalo Hump” (68, 69). Although literature documenting GC side effects is very abundant, the molecular mechanisms involved have not been completely elucidated, in part due to the complexity of the tissue specific effects of GCs.

Anti-inflammatory effects of GCs were historically associated with the monomeric form of GR, mainly due to the evidence that GR can bind and inhibit, and thus transrepresses the inflammatory transcription factor NFκB, downregulating the expression of pro-inflammatory cytokines (39, 70). The metabolic actions of GR were ascribed to the dimer, suggesting that drugs specific to monomeric, over dimeric GR would exhibit all beneficial anti-inflammatory effects without having negative side effects. A mouse model, in which GR dimerization is impaired (GR^dim^), has allowed several laboratories to show that GR dimerization is also required for the anti-inflammatory properties of GCs in several contexts, such as rheumatoid arthritis (71, 72), septic shock (73, 74), or inflammatory bowel disease (75). Interestingly however, the metabolic side effects of GCs are enhanced in the GR^dim^ mice. The loss of dimerization can drive increased insulin resistance and obesity, suggesting that the classical view of monomeric GR only being associated with the anti-inflammatory actions is not entirely correct (76). Therefore, both inflammatory and metabolic regulation by GCs may be driven by both the dimer and the monomer, depending on the cell type, the tissue, and the pathology considered.

## Glucocorticoids, Macrophages, and Tissue Repair

First investigations into the action of GCs on macrophages during tissue repair started a few decades ago. One of the side-effects of chronic GC exposure is the loss of bone mass (osteopenia/osteoporosis). Bone resorption, that is, the digestion of existing bone, is more efficient when highly specialized macrophages involved in bone remodeling, osteoclasts, are in direct contact with the bone. Resident tissue osteoclasts are derived from myeloid progenitor cells during development, however they are maintained throughout life by circulating blood monocytes fusing to existing osteoclasts in the bone (77). Osteoclasts treated with cortisol are more adherent to bone, more sensitive to RANKL, and release more calcium useable for bone resorption, enhancing the bone resorption process (78–80). GCs also increase osteoclastogenesis by driving the production of RANKL, the necessary factor for osteoclast differentiation, and downregulating osteoprotegerin, the decoy receptor for RANKL (81, 82). It was possible to prevent GC-induced osteoporosis by treating mice with a RANKL neutralizing antibody, further demonstrating that the effects of GCs on osteoclasts contribute to the bone loss that occurs during GC treatment (83). GCs can also have direct effects on osteoclasts. Using either mice deficient for GR in osteoclasts or 11BHSD2 overexpressing mice (where the GC inactivating enzyme is over-expressed in osteoclasts), it was confirmed that GCs act directly on osteoclasts to modulate bone density, in part by increasing the life span of osteoclasts (84, 85). Interestingly, chronic treatment with GCs decreases osteoclast life-span, suggesting a temporal effect (67, 86).

A mouse model based on the cre/loxP system was designed to specifically deplete GR in the myeloid lineage where the *cre* recombinase gene is located at the Lysozyme M locus. These so-called LysM^cre^;GR^fl/fl^ mice, delete GR in monocytes, macrophages and neutrophils. In a mouse model of contact hypersensitivity, the anti-inflammatory effects of GCs were shown to be mediated through GR in macrophages, rather than other tissues. Treatment of LysM^cre^;GR^fl/fl^ mice with GCs failed to repress the cytokines IL1-β, MCP1, MIP2, and IP10. In addition, GR^dim^ mice are also insensitive to GCs, indicating that GR dimerization, likely in macrophages, is required in this context (87). In a model of myocardial infarction, LysM^cre^;GR^fl/fl^ mice die earlier after infarction than wild-type animals with full expression of macrophage GR, probably due to the persistence of Ly6C^pos^ macrophages into the infarcted area, leading to a dysregulation of the resolution of inflammation and a defects in wound healing. This results in alteration of angiogenesis, abnormal production of TGFβ, decreased production of IL-1α and finally deregulation of myofibroblast differentiation leading to scar formation (88). Moreover, in a mouse model of inflammatory bowel disease, macrophages from LysM^cre^;GR^fl/fl^ animals show a defect in the acquisition of the anti-inflammatory status. After 10 days, IL-1β, and IL-6 expression is not repressed and expression of anti-inflammatory genes (CD163, CD206, and IL-10) is not induced, leading to a defect in tissue repair (89). Local availability of GCs also plays an important role in inflammation. The enzyme 11-β-hydroxysteroid dehydrogenase (type-1) (11bHSD1) catalyzes the conversion of the inactive cortisone to cortisol, enabling binding to GR and signaling. Myeloid specific knockouts of 11bHSD1, preventing endogenous GC signaling in macrophages and neutrophils, result in a more severe arthritis phenotype (90). This is however not limited to macrophages, inhibition of 11bHSD1 increases neutrophil recruitment during peritonitis (91).

Expansion of GC research into zebrafish models is still in the early stages, and so appears somewhat contradictory. No effect of the GC beclomethasone has been observed on the migratory capacity of macrophages toward the wounding area in an amputation model in zebrafish (92). However in a separate model of wounding, prednisolone reduced macrophage accumulation in both larvae and adults (93). This may be due to the different ligands used, as different ligands have previously been shown to have different transcriptional effects (51). Thus, in most tissue injuries, GC-GR axis appears to be a central pathway in macrophages to regulate the resolution of inflammation and to proceed to tissue repair after injury.

## Glucocorticoids and Macrophages—Cellular Aspects

### GCs Regulate Survival, Migration, and Proliferation of Macrophages

Maintenance of living immune cells in appropriate numbers is essential to modulate the inflammatory response, and GCs appear to play several roles in the regulation of macrophage life-span. GCs exert anti-apoptotic effects on macrophages: macrophages treated with dexamethasone are more resistant to lipopolysaccharide (LPS)-induced apoptosis (94). Similar results were obtained with other apoptotic stimuli (staurosporine, actinomycin D, or cyclohexine) where GC effects are mediated through ERK1/2 phosphorylation in an adenosine receptor A3-dependent-manner (95, 96). Moreover, macrophages treated with dexamethasone are smaller with less cytoplasmic extensions (97), which could be related to altered migratory capacity. The capacity of macrophages to move toward the injured area also shapes the inflammatory response. Macrophages treated with hydrocortisone (cortisol) show a decreased capacity to migrate *in vitro* (98, 99). *In vivo*, a similar effect was observed in a model of lung injury induced by bleomycin, where GCs inhibited macrophage infiltration into the lung (100). Studies using myeloid like cells and whole bone marrow preparations showed that GCs decrease proliferation of cells (including macrophages) *in vitro* (101, 102), but GC impact on proliferation has never been investigated on macrophage cultures. GR activation also has potent effects on nitric oxide (NO) production by macrophages. Initial studies in the J774a.1 macrophage cell line demonstrated that GCs suppress the induction of the NO-generating enzyme, nitric oxide synthase, thus controlling the level of NO produced by the cells in response to an inflammatory stimulus (103). Later studies however, showed that GCs are protective in a mouse model of stroke through increasing NO production in a non-genomic manner. By activating PI3K, GCs rapidly induce NO dependent vasodilation (104). The effects of GCs on NO production were further demonstrated to be dose dependent, with lower doses eliciting an increase in NO, while higher doses reducing the production of NO (105).

Thus, GCs promote macrophage survival in order to switch off inflammation and to sustain late phase of healing. In the following decades, studies have focused on the understanding of the molecular aspects of GC signaling pathways.

### GCs and Phagocytosis

During inflammation, damaged tissue produces cell debris, and releases cytoplasmic proteins into the environment due to cell lysis (106). Before tissue repair can start, debris must be cleared up (106). The clearing process is mainly performed by neutrophils, then macrophages, through phagocytosis of tissue debris, i.e., efferocytosis (106). Since phagocytosis is a major function of macrophages and is an essential trigger of their inflammatory switch (see above section “Macrophage phagocytosis and the resolution of inflammation”), the action of anti-inflammatory treatments on this process is of importance. GCs were detected very early to have an impact on phagocytic activity of macrophages (107). Later on, studies showed in *in vitro* models using a variety of particles (zymosan, heat-kill yeast, apoptotic neutrophils, latex beads, bacteria) that dexamethasone increases the phagocytic activity of monocytes/macrophages (95, 102, 108–115). Some of these studies have also shown, using a GR antagonist (RU486), that GC-dependent phagocytosis is also GR dependent (109, 110). The increased macrophage phagocytic activity by dexamethasone is annexin 1-FRP1 dependent (116). Annexin 1 belongs to the superfamily of annexin protein, which bind acidic phospholipids in the presence of Ca^2+^ (116). Annexin A1 is described to be a pro-resolving molecule during inflammation (117). Indeed, when the annexin receptor FRP1 is antagonized by the Boc1 compound or in annexin 1-null macrophages, dexamethasone loses its effect on phagocytosis (118).

On closer examination of the phagocytic process, it became clear that GCs induce the up-regulation of several membrane receptors, such as the scavenger receptor CD163, required to detect and bind haptoglobin, a product from hemoglobin degradation (111, 113, 114, 119). The mannose receptor CD206, required for the detection of specific oligosaccharides on the bacterial wall, is also upregulated in macrophages treated by GCs (120). Moreover, GCs upregulate the membrane receptor Mer tyrosine kinase (MerTK) (121), in a C/EBPβ dependent-manner (122). When *mertk* is silenced, dexamethasone-induced phagocytosis is reduced (121). MerTK belongs to the Tyro3, Axl, MerTK (TAM) family of tyrosine kinase receptor. It binds to phosphatidyl serine exposed on the surface of apoptotic cells (121, 122). MerTK is also responsible for the phagocytosis of protein S-opsonized apoptotic neutrophils by GC-treated macrophages (123). The other members of the TAM family do not seem to be necessary for GC-induced phagocytosis, as Tyro3 deficient, or Axl deficient mice are able to successfully clear apoptotic cells in response to GCs (124). Interestingly, in a model of serum-transfer induced arthritis, Axl, MerTK, and CD163 upregulation in macrophages requires GR function on synovial fibroblasts, indicating their regulation through cross-talk between local cells (72). Finally, GCs regulate the C/EBPβ-dependent expression of nuclear receptors (liver X receptor [LXR], retinoid X receptor α [RXRα] and peroxisome proliferator-activated receptor δ [PPARδ]), which are required for prolonged phagocytosis of macrophages (122). Thus, GCs act on several steps of phagocytosis and their effects are mediated through various signaling pathways.

## Glucocorticoids and Gene Expression in Macrophages—Molecular Aspects

Although the first effects of GCs on macrophages were reported in 1950, the literature about their specific effects on this cell type is not abundant (see section GCs on macrophages: expression of anti-inflammatory effectors). In 1950, Dougherty and colleagues showed in a model of local inflammation in mice that cortisone treatment reduces the number of macrophages in the inflamed area (125). In another model of skin inflammation induced by injection of turpentine, Spain et al. showed that cortisone inhibits the formation of granulation in the inflamed area (granulations corresponding to macrophages according to the authors) and a decrease of carbon particle phagocytosis when administrated early during the inflammatory response (107). However, the experiments done by Gell and Hinde on intraperitoneal macrophages exposed to bacteria showed that cortisone does not alter either the number of macrophages or their phagocytic capacity (126).

### GCs on Macrophages: Expression of Anti-inflammatory Effectors

It is well-known that macrophages can exert pleiotropic functions through the secretion of a variety of factors. Macrophages are highly versatile, and may secrete pro-inflammatory, anti-inflammatory, or other factors necessary at each step of the inflammatory response. GCs decrease the secretion of the pro-inflammatory cytokines TNFα (94, 127), IL-1, IL-6 in macrophages exposed to IFNγ (100, 113). Monocytes treated with GCs increase their secretion of IL-10 and TGFβ (128, 129) and express high levels of the anti-inflammatory membrane markers CD206 (120), CD163 (95, 111, 113, 114, 119, 130) and CD169 (95, 131). GC anti-inflammatory effects are partly mediated by Mitogen-activated protein kinase phosphatase-1 (MKP-1) in macrophages, as it was GC-driven inhibition of IL-6 expression was abrogated in MKP-1 deficient macrophages (132).

Furthermore, macrophages exposed to GCs secrete molecules which have direct functions on the extracellular matrix and therefore participate to matrix remodeling during the late phase of the inflammatory response. The production of elastase, collagenase and plasminogen activator (whose secretion is elevated in pro-inflammatory macrophages and which are required to degrade extracellular matrix) is reduced in macrophages treated with GCs (133, 134). On the contrary, macrophages exhibiting an alternatively activated status (i.e., IL-4 driven) secrete more fibronectin when treated with GCs, participating in matrix remodeling at the time of tissue repair (114, 135, 136).

### GC Action on Macrophages: Regulation of Gene Expression

GCs act through either the GR dimer or GR monomer, entirely depending on the gene regulated. For example, in dermatitis, GR dimerization is required to shut down the expression of the pro-inflammatory cytokines IL-1β and MCP-1 whereas TNFα downregulation induced by GCs does not require GR dimerization (87). GCs also modulate chromatin architecture, mainly closing down access to genes involved in inflammation, preventing access to other transcription factors (137, 138).

Importantly, the gene regulatory actions of GCs depend on the activation state of macrophages. Indeed, more than 10,000 genomic GR binding sites are induced by dexamethasone in resting macrophages with more than 5,400 known GR target genes, while in macrophages pre-treated with GCs, then LPS, there is a rewiring of GR binding, with 13,000 binding sites and more than 6,400 GR target genes identified (139). Furthermore, GCs regulate a different set of genes in macrophages activated with LPS or IFNγ indicating that genes are regulated by GCs are also dependent on the inflammatory stimulus (130). LPS stimulation also increases the ability of GR to bind DNA indicating that pro-inflammatory stimulation potentiates GR DNA binding, likely through the generation of more potential binding loci (138, 139). Oh et al. also demonstrated that pre-treatment compared to post-treatment of GCs with LPS results in a differential effect on gene regulation. The number and location of GR binding sites and p65 binding sites were different between the GC pre-treated cells and the cells treated with LPS first, then GCs (138). Furthermore, another GR partner, the Glucocorticoid Receptor-Interacting Protein (GRIP) 1, also known as nuclear receptor co-activator 2 (NCOA2) is required for the acquisition of the anti-inflammatory phenotype of macrophages (140). GRIP1 can be phosphorylated by Cyclin-Dependent Kinase 9 (CDK9) in a GR dependent-manner. Phosphorylated GRIP1, in association with GR, binds GREs to induce the expression of anti-inflammatory genes. However, phosphorylated GRIP1 is not observed in GR repressed sites such as of *IL1a* or *IL1b*, indicating that phosphorylated GRIP1 only acts on positive transcription of anti-inflammatory genes, and it is likely that the phosphorylation status of GRIP1 can modulate GR transcriptional activity (141). Our understanding of the role of GR as an anti-inflammatory transcription factor is still evolving, and with new technologies, the actions of GR will become clearer with time.

### The GC Effector GILZ in Macrophages

GC-mediated anti-inflammatory effects are known to be partly mediated through the regulation of the expression of specific proteins that in turn modulate inflammatory signaling. A very well-studied example is Glucocorticoid-Induced Leucine Zipper (GILZ). Originally found expressed in lymphoid tissues (thymocyte, spleen, lymph nodes) treated by dexamethasone (142), GILZ is a major regulator of GC effects in a variety of cells. GILZ was also found to be expressed by macrophages in liver and lung treated by dexamethasone (143). In the THP-1 macrophage cell line, dexamethasone induces *Gilz* mRNA expression after only 30 min of treatment (143). GILZ acts by binding the p65 subunit of the NFκB complex to shut down its activity (143). GILZ also inhibits the expression of the Toll like receptor 2 (TLR2), thus limiting the recognition of bacterial components and the associated inflammatory signaling (143). GCs however also enhance the expression of TLR2 in a cell-type specific manner (144, 145), suggesting that GILZ may act as a homeostatic brake on GC enhanced TLR2 signaling. Furthermore, GC-induced GILZ expression is strongly reduced in annexin A1 deficient macrophages, therefore preventing the downregulation of the pro-inflammatory cytokines IL-1, IL-6, and TNFα (146, 147). This regulation is not dependent of the annexin receptor FRP (146), thus, the exact mechanism by which annexin regulates *Gilz* expression remains to be elucidated.

## Conclusion

The effects of GCs on macrophages, especially in the broader context of resolution of inflammation during tissue repair, are not as well-understood as one would assume. GCs play key roles in the regulation of macrophage homeostatic functions, as well as the macrophage function as innate immunity cells. GR however, does not act alone. In association with several partners including other transcription factors (C/EBPβ, PPARs, NFκB) or proteins that modulate its activity (GRIP1), GR controls the functional properties of macrophages to resolve inflammation and tissue damage. Finally, GCs regulate the expression of a huge number of genes that are essential to relay their anti-inflammatory properties such as *Gilz* and *Annexin a*1. Despite 60 years of work on GCs, we are still discovering further molecular mechanisms that govern their actions. The role of the inflammatory context (138, 139) and species differences in GC mediated gene regulation (148) highlight that further investigation is necessary to decipher, for each situation, how GCs operate to regulate gene expression, and therefore control macrophage function.

## Author Contributions

TD, GC, RM, JT, and BC writing and editing of the manuscript.

### Conflict of Interest Statement

The authors declare that the research was conducted in the absence of any commercial or financial relationships that could be construed as a potential conflict of interest.
